# A High-Resolution Network with Strip Attention for Retinal Vessel Segmentation

**DOI:** 10.3390/s23218899

**Published:** 2023-11-01

**Authors:** Zhipin Ye, Yingqian Liu, Teng Jing, Zhaoming He, Ling Zhou

**Affiliations:** 1 Research Center of Fluid Machinery Engineering & Technology, Jiangsu University, Zhenjiang 212013, China; 2 Department of Mechanical Engineering, Texas Tech University, Lubbock, TX 79411, USA

**Keywords:** fundus image, retinal vessel segmentation, deep learning, high-resolution representation, strip attention module

## Abstract

Accurate segmentation of retinal vessels is an essential prerequisite for the subsequent analysis of fundus images. Recently, a number of methods based on deep learning have been proposed and shown to demonstrate promising segmentation performance, especially U-Net and its variants. However, tiny vessels and low-contrast vessels are hard to detect due to the issues of a loss of spatial details caused by consecutive down-sample operations and inadequate fusion of multi-level features caused by vanilla skip connections. To address these issues and enhance the segmentation precision of retinal vessels, we propose a novel high-resolution network with strip attention. Instead of the U-Net-shaped architecture, the proposed network follows an HRNet-shaped architecture as the basic network, learning high-resolution representations throughout the training process. In addition, a strip attention module including a horizontal attention mechanism and a vertical attention mechanism is designed to obtain long-range dependencies in the horizontal and vertical directions by calculating the similarity between each pixel and all pixels in the same row and the same column, respectively. For effective multi-layer feature fusion, we incorporate the strip attention module into the basic network to dynamically guide adjacent hierarchical features. Experimental results on the DRIVE and STARE datasets show that the proposed method can extract more tiny vessels and low-contrast vessels compared with existing mainstream methods, achieving accuracies of 96.16% and 97.08% and sensitivities of 82.68% and 89.36%, respectively. The proposed method has the potential to aid in the analysis of fundus images.

## 1. Introduction

Fundus imaging is a non-invasive, reproducible, and inexpensive method that shows retinal vessels and pathology [1]. In the medical domain, the morphological changes of retinal vessels, e.g., vessel diameter, branch angles, and branch lengths, can be used as clinical indicators for the detection and diagnosis of diabetes, hypertension, atherosclerosis, and other diseases [2]. In addition, the retinal vascular tree can serve as a unique identifier for identification systems in the social security domain, due to the unique morphology of this feature in individuals [3]. Retinal vessel segmentation is the process of determining from fundus images whether each pixel is a vessel or non-vessel pixel and is the preliminary step in objectively assessing retinal vasculature and quantitatively interpreting the morphometrics. Nevertheless, manual approaches to retinal vessel segmentation by trained experts are expensive, time-consuming, and laborious, especially in screening a large number of people. Furthermore, manual segmentation cannot guarantee segmentation performance because the results often vary from expert to expert due to their subjective segmentation. Therefore, the development of an automatic and high-precision method for retinal vessel segmentation is highly demanded. However, the retinal vascular tree presents an extremely complicated morphological structure and has many tiny vessels with a width of fewer than ten pixels or even one pixel, and are, therefore, generally difficult to distinguish from the background. Similarly, owing to uneven illumination and lesion regions, the contrast between blood vessels and non-vascular structures is relatively low. Because of these problems, it remains a challenging task to accurately segment retinal vessels from fundus images, especially tiny vessels and low-contrast vessels.

In 1989, Chaudhuri et al. became the first to deal with the problem of automatically segmenting retinal vessels [4]. Following this work, many methods have been proposed for retinal vessel segmentation, spurred by developments in digital image processing technology in recent decades [5]. Early studies based on various hand-crafted features, e.g., shape [6], color [7], and edge [4], usually exhibit low accuracy and poor robustness due to the features being shallow and insufficiently expressing semantic-rich information. Recently, deep learning methods, especially deep convolutional neural networks (DCNNs), have achieved superior results for many computer vision tasks, e.g., image classification [8], object detection [9], human pose estimation [10], and semantic segmentation [11]. Compared with conventional methods, DCNNs are able to automatically learn richer representations from raw input data and demonstrate superior segmentation performance [12]. In particular, Long et al. [13] proposed a novel end-to-end and pixel-to-pixel semantic segmentation network, called FCN, which introduces the most basic framework for natural image segmentation: the encoder–decoder structure. However, unlike the large number of natural image datasets available, the number of medical image datasets is relatively small because they are difficult to collect due to patient privacy and ethical issues. In this regard, Ronneberger et al. [14] proposed U-Net, an improvement on FCN, that could be trained with only a few images and still predict precise results. U-Net is a breakthrough advancement in deep learning in the field of medical image segmentation. In addition to its encoder–decoder structure, the success of U-Net is largely attributed to the skip connection between the encoder sub-network and the decoder sub-network, which combines multi-level features at different stages. As a general rule, the low-level features of shallow layers have abundant spatial details but lack sufficient semantic information, while the high-level features of deep layers have semantic-rich information but lose spatial details. It is an intuitive method that adopts the skip connection to fuse the spatial details of the encoder sub-network and the semantic information of the decoder sub-network.

Even though U-Net and its variants have achieved state-of-the-art results on many medical image segmentation tasks including kidney segmentation, pancreas segmentation, and liver segmentation, it is still not good enough to efficiently and effectively segment retinal vessels. In general, there are two main limitations. Firstly, consecutive down-sample operations in the encoder sub-network result in the loss of spatial information of tiny vessels and vessel edge information, and the final segmentation map cannot recover this lost information through skip-connections and up-sample operations in the decoder sub-network. Clinically, tiny vessels consisting of only several pixels provide an indispensable reference for the diagnosis of diseases like neovascular diseases. Therefore, we should pay more attention to the tiny vessels than to the thick vessels. Secondly, there exists a certain semantic gap between low-level features and high-level features in fundus images, especially in low-contrast regions. The vanilla skip connection introduces too much irrelevant redundant information, harming retinal vessel segmentation performance, especially with low-contrast vessels. It is essential to intelligently enhance vessel representations while suppressing background noise.

To tackle these problems and improve the segmentation precision of retinal vessels, we propose a novel network based on HRNet [15] and the self-attention mechanism [16]. The proposed network is implemented based on an HRNet-shaped architecture and integrates our newly designed strip attention module. The main contributions in this paper are summarized as follows:

(1) The proposed network retains high-resolution representations instead of employing the encoder–decoder architecture, and accordingly, learns high-resolution representations throughout the training process;

(2) A strip attention module is proposed to adaptively and effectively fuse multi-level features. The module can calculate the similarity between each pixel and all pixels in the same row and the same column, respectively, thereby selectively emphasizing salient features useful for a specific task while suppressing redundant irrelevant information;

(3) We empirically conduct experiments on the DRIVE and STARE datasets, achieving state-of-the-art results.

The rest of this paper is organized as follows. In Section 2, we introduce a brief literature review of the related works. Section 3 explains the proposed method in detail. Section 4 describes the details of the experiments. Section 5 presents the results and analysis. Discussions and conclusions of our work are given in Section 6 and Section 7, respectively.

## 2. Related Works

### 2.1. Deep Convolutional Neural Networks

To date, many segmentation networks based on the fully convolutional network (FCN) with the encoder–decoder (high-to-low and low-to-high in series) architecture have been proposed in the field of semantic segmentation. Among them, U-Net [14] and its variants have achieved remarkable performance in medical image segmentation including retinal vessel segmentation. For instance, DUNet [17] replaced standard convolutions with deformable convolutions because of the high complexity of the structures of retinal vessels. Zhang et al. [18] introduced new edge-aware flows into U-Net to make predictive outcomes more sensitive to vessel edge information. For multi-source vessel image segmentation, Yin et al. [19] designed a deep fusion network, called DF-Net, which is composed of multi-scale fusion, feature fusion, and classifier fusion. Li et al. [20] proposed a multi-task symmetric network, called GDF-Net, which consists of three typical U-Net-shaped sub-networks consisting of a global segmentation network branch, a detail enhancement network branch, and a fusion network branch. As an alternative to the encoder–decoder architecture, Guo [21] put forward a low-to-high segmentation architecture, called CSGNet, which first obtains low-resolution representations, and then learns high-resolution representations with the help of low-resolution representations. Recently, some studies [15,22] have demonstrated that learning high-resolution representations throughout the training process can preserve spatial details of tiny vessels and vessel edge information, which is beneficial to segmenting tiny vessels and locating vessel boundaries. A representative method is HRNet, which was originally proposed for human pose estimation and used for other position-sensitive vision tasks [15]. HRNet maintains high resolutions from input data to final outcomes without the requirement of restoring high resolutions and generates semantic-rich high-resolution representations via repeatedly exchanging information from multi-resolution features. Motivated by HRNet, Lin et al. [22] proposed a novel high-resolution representation network with a multi-path scale, called MPS-Net. In MPS-Net, there are three paths with different resolutions, in which the main path maintains high resolutions throughout the entire process, while two branch paths with low-resolution representations are added to the main path in parallel. In this paper, an HRNet-shaped network is used as the basic network due to its superior performance on retinal vessel segmentation.

### 2.2. Self-Attention Modules

Generally speaking, humans can analyze and understand complex scenes naturally and effectively. Motivated by this observation, attention mechanisms [23,24] were introduced into deep learning in order to dynamically adjust the weight of feature maps. In particular, Vaswani et al. [16] proposed a self-attention mechanism with the aim of acquiring the long-range dependencies of timing signals, which facilitates machine translation and natural language processing. Then, Wang et al. [25] introduced the self-attention mechanism into computer vision to obtain long-range dependencies via non-local operations. Based on the self-attention mechanism, Fu et al. [26] presented DANet for scene segmentation, which includes a position-attention module to focus on the relationship in the spatial dimension and a channel-attention module to pay attention to the interdependencies in channel dimensions. However, the self-attention mechanism needs to generate a huge attention matrix, whose complexity is $𝒪\left(\left(H\times W\right)\times \left(H\times W\right)\right)$, where H×W denotes the resolution of the input feature map, which seriously limits its practical applicability. Therefore, several variants of the self-attention mechanism have been proposed to reduce computational complexity. For instance, Huang et al. [27] viewed the self-attention operation as a graph convolution and utilized several sparsely connected graphs instead of the densely connected graph generated by the original self-attention mechanism. To do so, Huang et al. introduced a criss-cross attention module, whose weight is H+W−1 not H×W, reducing the computational complexity from $𝒪\left(\left(H\times W\right)\times \left(H\times W\right)\right)$ to $𝒪\left(\left(H\times W\right)\times \left(H+W-1\right)\right)$. In addition, Li et al. [28] regarded the self-attention mechanism in terms of an expectation–maximization manner to obtain a much more compact set of bases, reducing the computational complexity from $𝒪\left(\left(H\times W\right)\times \left(H\times W\right)\right)$ to $𝒪\left(\left(H\times W\right)\times K\right)$, where K represents the number of the compact bases. Li et al. [29] designed a lightweight dual-direction attention block, generating the attention matrix with computational complexity of $𝒪\left(H\times W\right)$ via horizontal and vertical pooling operations. However, these existing variants are insufficient for retinal vessel segmentation, as they fail to focus on the characteristics of vessel structures. In this paper, an efficient strip attention module is proposed with better alignment with structures.

## 3. Methodology

### 3.1. Overview of the Network Architecture

**Figure 1:**
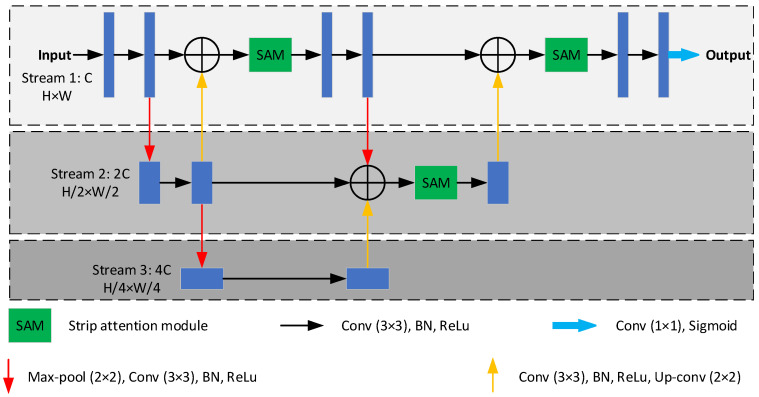
The architecture of the proposed network. (⊕ is broadcast element-wise addition).

As shown in Figure 1, the proposed network is implemented based on an HRNet-shaped architecture rather than the architecture of high-to-low and low-to-high in series and consists of three streams including a high-resolution main stream, a medium-resolution branch stream, and a low-resolution branch stream. In the main stream, the resolution is H×W from input data to final outcomes, and the depth (the number of channels) remains C, changed by 3×3 convolutional layers. To obtain a larger receptive field and richer semantic information, the two branch streams are gradually conducted into the main stream in parallel. The resolutions of the medium-resolution stream and the low-resolution stream are H/2×W/2 and H/4×W/4, respectively, and the corresponding depths are 2C and 4C, respectively. Considering that retinal vessels vary greatly in terms of width, from one pixel to twenty pixels, multi-level fusion is repeated several times to exchange information across multi-resolution representations. What is more, to overcome the semantic gap issue of multi-level features and further enhance the fusion effect, we replace the vanilla skip connection with our proposed strip attention module.

Stacking small kernel convolutional layers can lead to a large receptive field, for example, two stacking 3×3 convolutional layers yield a receptive field of a standard 5×5 convolutional layer. Based on this characteristic, we employ deeply stacking 3×3 convolutional layers to model the representations of the proposed network, which significantly decreases the number of parameters and GPU memory to ease the training process. Meanwhile, we introduce batch normalization and nonlinear activation ReLU after every convolutional layer to reduce the change in the distributions of internal nodes of the proposed network and accelerate the training process by a significant margin. At the end of the proposed network, we utilize a convolutional layer and a sigmoid layer to obtain the final segmentation map.

### 3.2. Strip Attention Module

The original skip connection is used to combine multi-level feature maps with different scale information directly via concatenation or addition, but it is a native method that cannot efficiently and effectively merge representations. To deal with this issue, the self-attention mechanism may be a feasible solution. Taking a 2-D image as an example, the whole process of the self-attention mechanism [16,25,30] is as follows. Given a feature map $X\in {\mathbb{R}}^{C\times H\times W}$, where C, H, and W represent the depth, height, and width, respectively, the self-attention mechanism firstly feeds the map into a linear projection and a reshape operation to generate three branches: Q, K, and V, where $\left\{Q,K\left.,V\right\}\right.\in {\mathbb{R}}^{C\times N}$, N=H×W. Then, the self-attention mechanism can be formulated as

(1) $$A=\mathrm{Soft}max\left(Q⊗K^{T}\right)$$

(2) Z=ReshapeV⊗AT

where ⊗ is matrix multiplication; $K^{T}$ is the transpose of K; $A^{T}$ is the transpose of the self-attention matrix $A\in {\mathbb{R}}^{N\times N}$; and $Z\in {\mathbb{R}}^{C\times H\times W}$ is the output.

**Figure 2:**
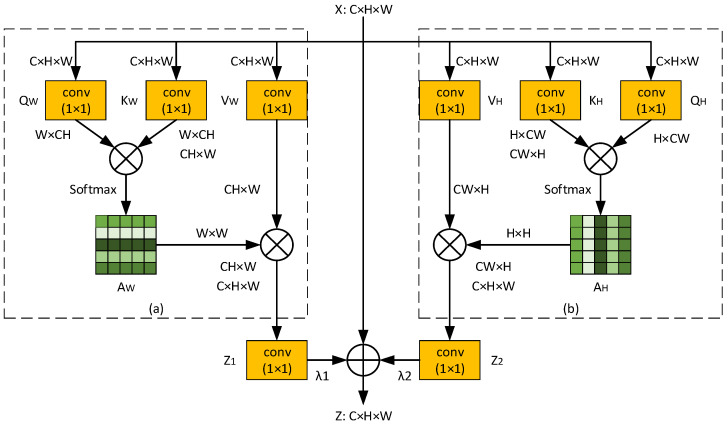
Illustration of the strip attention module. (**a**) Horizontal attention mechanism; (**b**) Vertical attention mechanism. (⊗ is matrix multiplication, and ⊕ is broadcast element-wise addition).

As shown in the process of the self-attention mechanism, a self-attention matrix is generated to calculate the similarity between each pixel and all pixels. As the self-attention matrix has a quadratic complexity of 𝒪($\left(N^{2}\right)$) and practically suffers from high computational and memory costs, the application of self-attention mechanisms is limited in real-time applications. At the same time, we note that vessels are thin and elongated in structure, and as such, the self-attention matrix may not be the perfect solution. That is to say, we need to pay more attention to the similarity of vessel pixels rather than all pixels. Therefore, we introduce a strip attention module based on the self-attention mechanism, as shown in Figure 2. The strip attention module consists of a horizontal attention mechanism (see Figure 2a) and a vertical attention mechanism (see Figure 2b) to obtain long-range dependencies in the horizontal and vertical directions, respectively. To be specific, the output feature map $Z_{1}\in {\mathbb{R}}^{C\times H\times W}$ of the horizontal attention mechanism and the output feature map $Z_{2}\in {\mathbb{R}}^{C\times H\times W}$ of the vertical attention mechanism are both fed into a 1×1 convolutional layer followed by batch normalization and nonlinear activation ReLU. Finally, we perform a broadcast element-wise addition with the input feature map $X\in {\mathbb{R}}^{C\times H\times W}$ to obtain the final output feature map $Z\in {\mathbb{R}}^{C\times H\times W}$, an approach that is similar to residual learning [31], to alleviate the notorious problem of vanishing/exploding gradients and aid the training process.

(3) $$Z=X+\lambda_{1}\times Z_{1}+\lambda_{2}\times Z_{2}$$

where $\lambda_{1}$ and $\lambda_{2}$ are both learnable parameters initialized as 0 to gradually assign more weight.

(a) Horizontal attention mechanism: As shown in Figure 2a, given a feature map $X\in {\mathbb{R}}^{C\times H\times W}$, we first employ three 1×1 convolutional layers followed by batch normalization and nonlinear activation ReLU on X to generate three branches: $Q_{W}$, $K_{W}$, and $V_{W}$, where $\left\{Q_{W},K_{W}\left.,V_{W}\right\}\right.\in {\mathbb{R}}^{C\times H\times W}$. Then, we reshape $Q_{W}$ and $K_{W}$ to $R^{W\times CH}$, where CH=C×H. Next, we perform a matrix multiplication between $Q_{W}$ and the transpose of $K_{W}$, and apply a softmax function to calculate the horizontal attention matrix $A_{W}\in {\mathbb{R}}^{W\times W}$:

(4) $$A_{W}=\mathrm{Soft}max\left(Q_{W}⊗K_{W}{}^{T}\right)\;{\left(A_{W}\right)}_{i,j}=\frac{exp\left({\left(Q_{W}\right)}_{i}•{\left(K_{W}{}^{T}\right)}_{j}\right)}{\sum_{i=1}^{W}exp\left({\left(Q_{W}\right)}_{i}•{\left(K_{W}{}^{T}\right)}_{j}\right)}$$

where ⊗ is matrix multiplication, ● is element-wise multiplication, and ${\left(A_{W}\right)}_{i,j}$ is an element of $A_{W}$ that measures the $i_{th}$ position’s impact on the $j_{th}$ position in the same row. The higher the value of ${\left(A_{W}\right)}_{i,j}$, the more similar $i_{th}$ and $j_{th}$ are in the same row, and vice versa. Meanwhile, we reshape $V_{W}$ to $R^{CH\times W}$. Finally, we perform a matrix multiplication between $V_{W}$ and the transpose of $A_{W}$, and reshape the result to ${\mathbb{R}}^{C\times H\times W}$, which is the output feature map of the horizontal attention mechanism. 

(b) Vertical attention mechanism: As shown in Figure 2b, similar to the horizontal attention mechanism, we first feed the input feature map X into three 1×1 convolutional layers followed by batch normalization and the nonlinear activation ReLU to yield three branches: $Q_{H}$, $K_{H}$, and $V_{H}$, where $\left\{Q_{H},K_{H}\left.,V_{H}\right\}\right.\in {\mathbb{R}}^{C\times H\times W}$. Then, we reshape $Q_{H}$ and $K_{H}$ to $R^{H\times CW}$, where CW=C×W. Next, we perform a matrix multiplication between $Q_{H}$ and the transpose of $K_{H}$, and apply a softmax function to count the vertical attention matrix $A_{H}\in {\mathbb{R}}^{H\times H}$:

(5) $$A_{H}=\mathrm{Soft}max\left(Q_{H}⊗K_{H}{}^{T}\right)\;{\left(A_{H}\right)}_{i,j}=\frac{exp\left({\left(Q_{H}\right)}_{i}•{\left(K_{H}{}^{T}\right)}_{j}\right)}{\sum_{i=1}^{W}exp\left({\left(Q_{H}\right)}_{i}•{\left(K_{H}{}^{T}\right)}_{j}\right)}$$

where ⊗ is matrix multiplication, ● is element-wise multiplication, and ${\left(A_{H}\right)}_{i,j}$ is an element of $A_{H}$ that measures the $i_{th}$ position’s impact on the $j_{th}$ position in the same column. The higher the value of ${\left(A_{H}\right)}_{i,j}$, the more similar $i_{th}$ and $j_{th}$ are in the same column, and vice versa. Meanwhile, we reshape $V_{H}$ to $R^{CW\times H}$. Finally, we perform a matrix multiplication between $V_{H}$ and the transpose of $V_{H}$, and reshape the outcome to ${\mathbb{R}}^{C\times H\times W}$, which is the output feature map of the vertical attention mechanism.

### 3.3. Loss Function

Due to the advantage of the binary cross-entropy loss $L_{BCE}$, it is widely used in semantic segmentation and defined as follows:

(6) $$L_{BCE}=-\frac{1}{N}\sum_{i=1}^{N}\left[y_{i}logp_{i}+\left(1-y_{i}\right)log\left(1-p_{i}\right)\right]$$

where $y_{i}$ denotes the true value of the position $i_{th}$, $p_{i}$ denotes the corresponding prediction value of the model, and *N* denotes the number of total pixels. As shown in Equation (6), the binary cross-entropy loss is the average loss of total pixels, and the contribution of each pixel is equal. We believe that the segmentation results would deviate towards the background pixels if we directly use the binary cross-entropy loss, due to the class imbalance problem that the number of the vessel pixels is small while the background pixels account for a large proportion of the entire fundus image. To alleviate the above problem, we introduce the Dice loss [32] as a regular term. The Dice loss function $L_{Dice}$ is defined as follows:

(7) $$L_{Dice}=1-\frac{2\sum_{i=1}^{N}y_{i}p_{i}+\varepsilon }{\sum_{i=1}^{N}y_{i}+\sum_{i=1}^{N}p_{i}+\varepsilon }$$

where $y_{i}$ denotes the true value of the position $i_{th}$, $p_{i}$ denotes the corresponding prediction value of the model, *N* denotes the number of total pixels, and ε denotes a small constant. ε is set to 1 × 10^−5^ in our experiments. The Dice loss function is based on the Dice coefficient, which can deal with the problem of imbalance between positive and negative samples. Therefore, we adopt a joint loss function $L_{Jo\mathrm{int}}$ to optimize the training process, which is calculated as follows:

(8) $$L_{Jo\mathrm{int}}=L_{BCE}+L_{Dice}$$

## 4. Experiments

### 4.1. Datasets

The proposed method is trained and tested on the DRIVE and STARE datasets, which are public and standard datasets for retinal vessel segmentation. As shown in Table 1, as the image formats of DRIVE and STARE are different, we convert all images to PNG images to facilitate the analysis. Given that the field of view (FoV) masks are not given on STARE, we manually generate the corresponding masks as described in [17] for the consistency of experimental results.

DRIVE: The DRIVE (Digital Retinal Images for Vessel Extraction) dataset [33], obtained from a diabetic retinopathy screening program in the Netherlands, contains 40 images with a resolution of 565×584, and is the most widely used public dataset to measure the performance of retinal vessel segmentation. There are 7 images with signs of mild early diabetic retinopathy and 33 images without any sign of diabetic retinopathy. We adopt the official data division, utilizing 20 images for model construction and the other 20 images for evaluation.

STARE: The STARE (Structured Analysis of the Retina) dataset [34] consists of 20 images, with half the images containing varying degrees of pathology. As pathological regions may obscure or confuse the blood vessel appearance in varying portions of the image, STARE is an extremely challenging dataset. The plane resolution of the fundus image is 700×605. Since no official split between the training set and test set is provided on the STARE dataset, we employ leave-one-out cross-validation [17,19], whereby every image is iteratively tested by the segmentation model trained by the remaining 19 images.

### 4.2. Fundus Image Preprocessing and Patch Extraction

**Figure 3:**
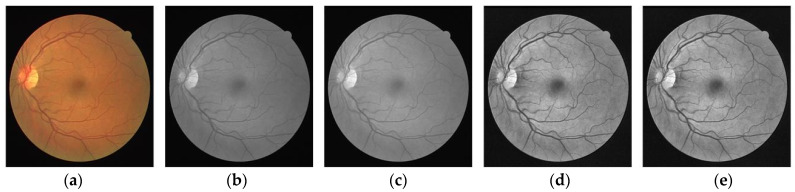
Preprocessing results. (**a**) Original image; (**b**) Grayed image; (**c**) Normalized image; (**d**) Image after CLAHE operation; (**e**) Image after Gamma correction.

Considering the low contrast of fundus images, we employ some preprocessing strategies. First, we transform the fundus images from RGB to gray. Following this, image normalization, contrast-limited adaptive histogram equalization (CLAHE) [35], and gamma transformation are sequentially utilized. As shown in Figure 3, the image quality is dramatically ameliorated and the contrast between vessel pixels and non-vessel pixels is effectively enhanced to a certain extent.

**Figure 4:**
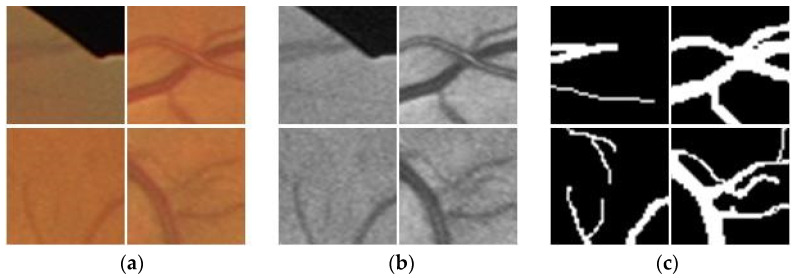
Typical 48 × 48 patches. (**a**) Original image patches; (**b**) Input image patches; (**c**) Corresponding label patches.

Because of the small number of images in the DRIVE and STARE datasets, we utilize a random crop operation after image preprocessing for data augmentation in order to mitigate the overfitting problem in the training process. Taking computational complexity and surrounding local features into account, we empirically set the plane resolution of the patch to 48×48 (see Figure 4). During the training stage, 1000 patches are randomly selected from each fundus image from the DRIVE and STARE datasets, in which 10% of the patches are used as the validation set for selecting the optimal weight of the training model. Therefore, the DRIVE dataset has 18,000 patches as the training set and 2000 patches as the validation set; while the STARE dataset has 17,100 patches as the training set and 1900 patches as the validation set.

### 4.3. Implementation Details

The implementation of the proposed network is based on the PyTorch platform (torch == 2.0.1 + cu118), and all experiments are built with an NVIDIA RTX 2080Ti graphics card (produced by NVIDIA in Santa Clara, CA, USA). We set the batch size to 64 according to the memory of the GPU card and the size of the model. We employ the initialization method as introduced in [31] to initialize the weights of all layers. During the training stage, we utilize the Adam algorithm as an optimization method, in which the initial learning rate and the weight decay are set to 1 × 10^−3^ and 1 × 10^−4^, respectively. In addition, we halve the current learning rate if the validation loss does not decrease for 5 consecutive epochs. If the validation loss does not decrease for 20 consecutive epochs, the model stops training.

**Figure 5:**
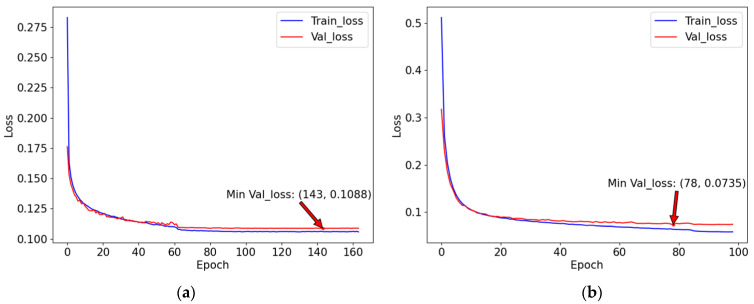
Loss curves. (**a**) DRIVE; (**b**) STARE.

Figure 5 shows loss curves for the proposed network on the DRIVE and STARE datasets in the training process, which show that the proposed network is easy to train. As shown in Figure 5, the optimum model is selected based on the minimum validation loss.

### 4.4. Evaluation Criteria

To comprehensively evaluate the proposed method, we quantitatively analyze the experimental results in terms of accuracy (ACC), sensitivity (SE), specificity (SP), and F1-score (F1), which have the following formulas:

(9) $$ACC=\frac{TP+TN}{TP+TN+FP+FN}$$

(10) $$SE=\frac{TP}{TP+FN}$$

(11) $$SP=\frac{TN}{FP+TN}$$

(12) $$F1=\frac{2TP}{2TP+FP+FN}$$

where TP (true positive) denotes a vessel pixel with correct classification; TN (true negative) denotes a non-vessel pixel with correct classification; FP (false positive) denotes that a non-vessel pixel is incorrectly predicted to be a vessel pixel; and FN (false negative) denotes that a vessel pixel is incorrectly predicted to be a non-vessel pixel. Additionally, AUC is also employed, which denotes the area under the receiver operating characteristic curve. Note that we only calculate the pixels inside the FoV.

## 5. Results and Analysis

We systematically compare the segmentation performance of the proposed network with U-Net and the baseline via a series of experiments on the DRIVE and SATRE datasets. It should be noted that the baseline is obtained by removing the strip attention module in the proposed network rather than by using the original HRNet by Wang et al. [15].

### 5.1. Experiments on DRIVE

Table 2 shows the comparison results of U-Net, the baseline, and the proposed network in terms of ACC, SE, SP, F1, and AUC on the DRIVE dataset. As shown in Table 2, the baseline obtains higher values compared with U-Net on key indicators such as ACC, SE, and AUC, while SP and F1 of the baseline are slightly worse than U-Net. In particular, the baseline achieves a significant improvement of 6.79% on SE, from 75.37% to 82.16%. As can be seen from the third and fourth rows of Table 2, the proposed method with the help of the strip attention module surpasses the baseline in terms of all metrics. Meanwhile, the ACC, SE, SP, F1, and AUC of the proposed method are increased by 0.85%, 7.31%, 0.27%, 2.85%, and 1.09%, respectively, compared with U-Net.

**Figure 6:**
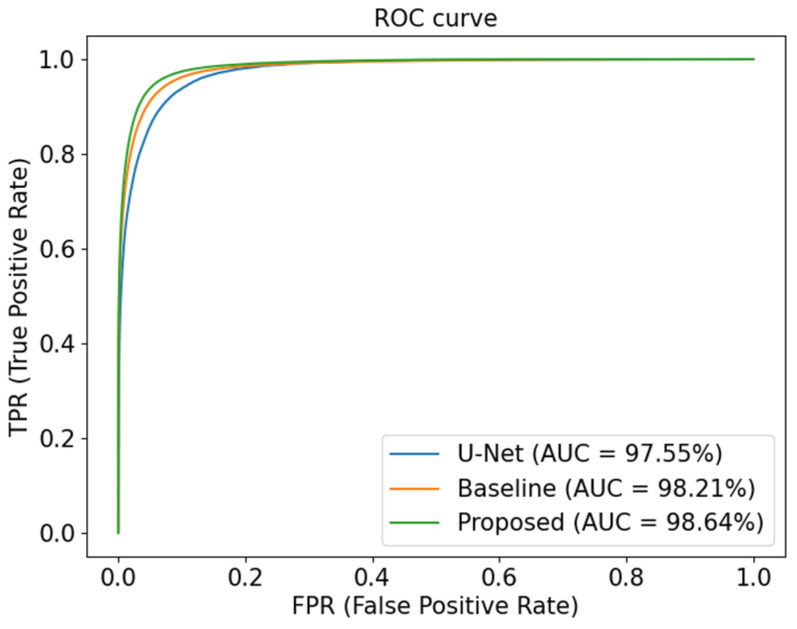
ROC curves of different models on DRIVE.

We also employed ROC curves to further evaluate the capability of different models on the DRIVE dataset, as shown in Figure 6. The closer the ROC curve is to the top-left border, the more accurate a model is. We can clearly observe that the proposed network outperforms other models on the DRIVE dataset, as shown by the AUC score.

**Figure 7:**
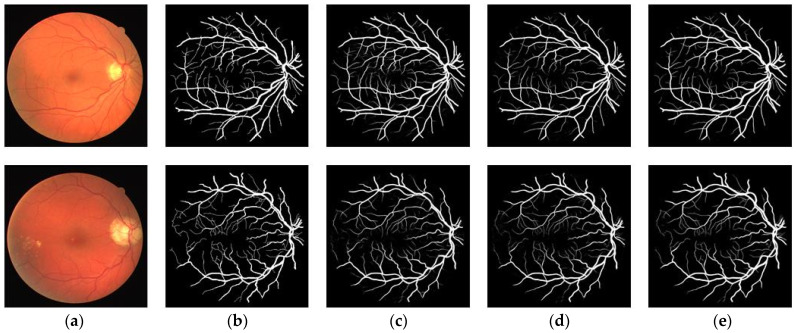
Some typical visualization results for different methods on DRIVE. (**a**) Original image; (**b**) Ground truth; (**c**) U-Net; (**d**) Baseline; (**e**) Proposed.

**Figure 8:**
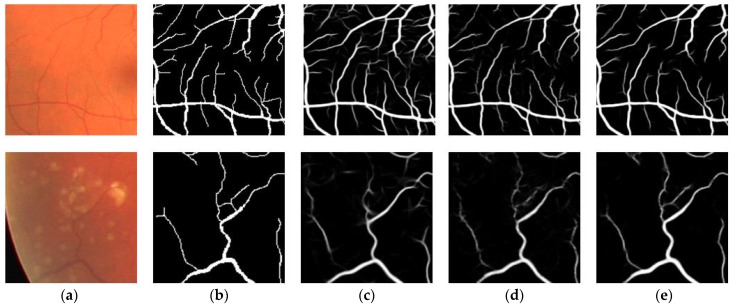
Visualization results of details on DRIVE. (**a**) Original image; (**b**) Ground truth; (**c**) U-Net; (**d**) Baseline; (**e**) Proposed.

In addition to the quantitative analysis, a qualitative analysis was also performed on the DRIVE dataset. Figure 7 shows the overall segmentation results of the three models on the DRIVE dataset, which include the entire retinal vessel structures. In Figure 7, the first row of images is from a healthy person while the second row contains fundus images with lesion areas. In order to further observe the details of the segmentation results, Figure 8 is presented to show the local magnification view of Figure 7. As shown in Figure 7 and Figure 8, all three methods can accurately segment thick vessels from fundus images. However, U-Net struggles to accurately detect tiny vessels consisting of only a few pixels and low-contrast vessels, especially in lesion areas. In contrast, the baseline can detect some tiny vessels, while, with the help of the strip attention module, the proposed model is able to distinguish more tiny vessels and deal with the lesion areas.

### 5.2. Experiments on STARE

Table 3 gives the comparison experiment results on the STARE dataset. It can be seen from Table 3 that U-Net achieves the highest SP score with 98.67%, which means that U-Net can better classify non-vessel pixels compared with the baseline and the proposed method. As the STARE dataset contains many lesion regions with some non-vessels that are extremely similar to vessel pixels, the baseline and proposed networks incorrectly predicted these pixels as vessel pixels because they pay too much attention to vessels due to maintaining high resolutions. For retinal vessel segmentation, SE is able to more objectively evaluate the segmentation performance than SP due to the highly imbalanced ratio between vessel pixels and non-vessel pixels. As shown in Table 3, it is worth noting that the baseline achieves a significant improvement of 11.35% in SE score compared with U-Net, and the proposed method obtains a significant improvement of 4.74% in SE score compared with the baseline. As for F1, the proposed method has the best value, increasing from 80.89% of U-Net and 84.23% of the baseline to 98.47%. At the same time, the ACC and AUC of the baseline are slightly increased by 0.36% and 0.78%, respectively, compared with U-Net, while slightly decreased by 0.44% and 0.79%, respectively, compared with the proposed method.

**Figure 9:**
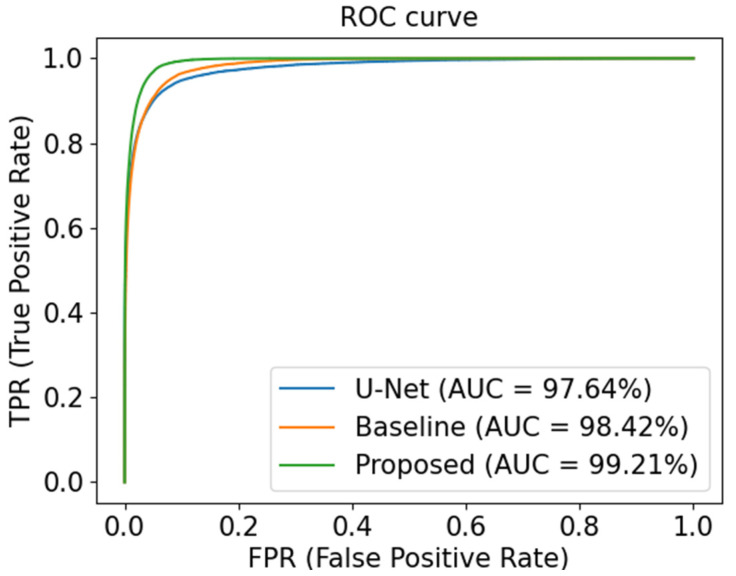
ROC curves of different models on STARE.

As shown in Figure 9, we also assess the three models on the STARE dataset by using ROC curves, which show that the segmentation performance of the proposed network is superior to that of its competitors.

**Figure 10:**
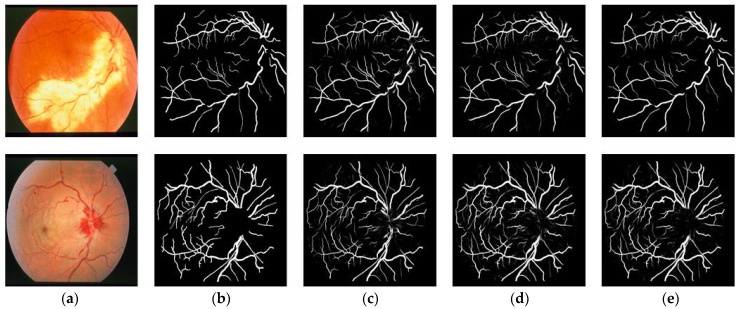
Some typical visualization results for different methods on STARE. (**a**) Original image; (**b**) Ground truth; (**c**) U-Net; (**d**) Baseline; (**e**) Proposed.

**Figure 11:**
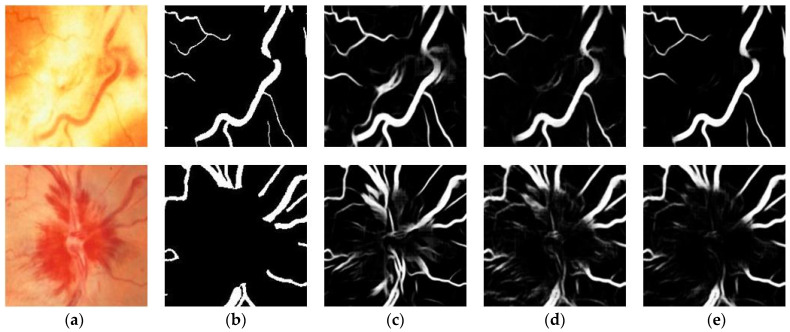
Visualization results of details on STARE. (**a**) Original image; (**b**) Ground truth; (**c**) U-Net; (**d**) Baseline; (**e**) Proposed.

Figure 10 shows the overall segmentation results of the three models on the STARE dataset, in which the first row is fundus images with plaques and the second row is fundus images with hemorrhages. As shown in Figure 10, plaques in the fundus image result in obvious differences between adjacent regions, while hemorrhage pixels are extremely similar to vessel pixels. These factors make retinal vessels, especially low-contrast capillaries, hard to segment. In order to more intuitively observe the segmentation performance, Figure 11 visualizes challenging patches selected in Figure 10 for experimental analysis. We observe that U-Net produces too many false positive pixels to serve as a general benchmark for retinal vessel segmentation. In contrast, based on learning high-resolution representations, the baseline focuses more on vessel edge presentation and surpasses U-Net. Meanwhile, the proposed model has a clear background and fewer artifacts compared with the other two methods, indicating that the proposed model achieves desirable segmentation results on tiny vessels and low-contrast vessels. 

### 5.3. Cross-Training Experiments

Compared with unsupervised methods, a major shortcoming of deep learning methods is that they are extremely reliant on the quality of training data, such that the segmentation performance may deteriorate if a well-trained model is applied to another dataset. In this section, we perform cross-training experiments (testing a model trained on one dataset on another dataset) on the DRIVE and STARE datasets to evaluate the extendibility and generalization of the proposed method. Table 4 shows the experimental results of the proposed method, other deep learning methods including those of Jin et al. [17] and Li et al. [20] and other unsupervised methods including those of Fathi et al. [36] and Zhao et al. [37]. Although the proposed method reduces the segmentation performance to a certain extent (see Table 2 and Table 3), it is still satisfactory compared with other comparison methods. When using the well-trained model on the STARE dataset to test the DRIVE dataset, the proposed model obtains the highest SE score of 81.47% among all comparison methods and achieves a significant improvement of 3.27% in SE score compared with the second-highest result. In addition, it also achieves state-of-the-art performance in terms of ACC, SP, and AUC. When we train the model on the DRIVE dataset and then test the STARE dataset, our proposed model achieves the top performance in terms of ACC, SE, SP, and AUC, with scores of 96.23%, 84.52%, 98.17%, and 97.92%, respectively. Possible explanations for the slight performance difference might be that the DRIVE dataset mainly contains tiny vessels and clearer images, while the STARE dataset mainly contains thick vessels and more pathological images.

## 6. Discussion

### 6.1. Comparison with Other Methods

Recent years have seen a renewal of interest in image segmentation based on DCNNs. In particular, U-Net has shown a promising segmentation performance on medical images. However, there are still some shortcomings with U-Net for retinal vessel segmentation, such as the loss of detail caused by consecutive down-sample operations and insufficient fusion caused by vanilla skip connections. Inspired by the architecture of HRNet and the self-attention mechanism, in this paper, we propose a novel method to deal with misleading diagnoses caused by the above problems. To show the superiority of the proposed method, we compare it with other proposed deep learning methods on the DRIVE and STARE datasets, as shown in Table 5 and Table 6. Note that [38] does not report F1 scores. 

In Table 5, the best indicators of ACC, SE, SP, F1, and AUC achieved by methods reported in the literature are 96.22%, 83.61%, 98.52%, 83.15%, and 98.59%, respectively, while the performance indicators of our proposed method are 96.16%, 82.68%, 98.47%, 84.27%, and 98.64%, respectively. It is worth emphasizing that the proposed method achieves a new state-of-the-art performance in terms of F1 and AUC on the DRIVE dataset, which proves the high ability of the model to distinguish between vessels and non-vessels. To be specific, the proposed method outperforms high-resolution networks, such as [21,22,39]. Although the proposed method is slightly worse than MPS-Net [22] in terms of SE, the proposed method achieves higher scores on other metrics including ACC, SP, F1, and AUC. Meantime, the proposed method also has superior performance compared to the U-Net-shaped models with an attention mechanism detailed in references [20,38,40,41,42,43]. To be specific, the F1 score of GDF-Net [20] is over 1% lower than that of the proposed method. In addition, GDF-Net has shortcomings in the training process due to it consisting of three typical U-Net-shaped sub-networks.

As can be seen in Table 6, among all comparison methods, the best indicators of ACC, SE, SP, F1, and AUC achieve scores of 97.10%, 85.66%, 99.57%, 85.42%, and 99.10%, respectively, while the ACC, SE, SP, F1, and AUC scores obtained by the proposed method are 97.08%, 89.36%, 98.47%, 87.85%, and 99.21%, respectively. At the same time, the proposed method obtains the best results in terms of SE, F1, and AUC compared the best method among all comparison methods. In particular, it is worth noting that the proposed method achieves a significant improvement of 3.70% in SE score and 2.43% in F1 score compared with the second-highest result, indicating that the proposed method can correctly predict more vessel pixels. Although GDF-Net [20], recently proposed by Li et al., achieves the highest SP score, its scores are lower than those of the proposed method in terms of the other evaluation metrics. Meanwhile, the proposed method achieves higher scores in all indicators compared with MAGF-Net [43], which also supports the superiority of the proposed method.

### 6.2. Advantages

Retinal vessels contain abundant biological information and can be used as a feature for computer-aided systems. However, fundus images usually present some challenging elements such as uneven illumination, weak texture, inconsistent vascular thickness, and complicated vascular structure, which readily result in pseudo results for retinal vessel segmentation. In addition, the complicated pathological manifestations in fundus images pose a great challenge to the accurate segmentation of retinal vessels from lesion regions. In order to improve the segmentation precision of retinal vessels, a novel HRNet-shaped network with strip attention modules is proposed. Instead of the encoder–decoder architecture used by models such as U-Net, the proposed network adopts a high-resolution network as the baseline network, maintaining high-resolution representations throughout the whole process, which contributes to obtaining spatial details of tiny vessels and vessel edge information. On this basis, a strip attention module is proposed to reduce the semantic gap issue between multi-level features and enhance the feature fusion process, highlighting salient features while suppressing irrelevant information. The strip attention module consists of a horizontal attention mechanism and a vertical attention mechanism, which can capture long-range dependencies in the horizontal and vertical directions. The advantage of the strip attention module is twofold. First, the strip attention module has better alignment with the shape of retinal vessels compared to the original self-attention module. Second, unlike the original self-attention matrix of H×W weights, the attention matrix of the horizontal attention and vertical attention mechanisms has W weights and H weights, respectively. The higher the resolution of the input feature map, the lower the computation and memory costs of the strip attention module compared to the original self-attention module. 

To test the segmentation performance of the proposed method on retinal vessels, especially low-contrast capillaries, we performed a series of experiments on the DRIVE and STARE datasets. Quantitative and qualitative analyses show that the proposed method can accurately and effectively segment retinal vessels from fundus images, even outperforming other existing methods to some extent. Even in lesion regions, the segmentation results of the proposed method are very close to the ground truth. Extensive cross-training experiments are used to further assess the extendibility and generalization of the proposed method. The promising segmentation performance reveals that the proposed model has the potential to be deployed on portable devices.

### 6.3. Limitations

Although the proposed method has achieved remarkable performance on retinal vessel segmentation, it still possesses some limitations. First, we only take the horizontal and vertical directions into account in the strip attention module, which may result in some fractured vessels, as vessels are oriented in many directions. Second, with the proposed method, the average processing time (including inference and I/O) for one fundus image from the DRIVE and STARE datasets is almost 1.86 s and 2.47 s, respectively. In addition, the process of patch extraction and prediction reconstruction is also memory intensive. In general, two main aspects need to be addressed. One is that even though we utilize random crop operation to augment the number of the DRIVE and STARE datasets, this does not actually increase the number of images in the datasets. In the follow-up study, we will explore Generative Adversarial Networks (GANs) to augment the datasets artificially. Another aspect that needs to be addressed is that the resolution of the DRIVE and STARE datasets is far from that of current fundus image acquisition systems. If the input feature maps have very high resolution, the memory and operations of the proposed network will increase dramatically, mainly because of the strip attention module. Therefore, we will continue this research by focusing on a more lightweight model based on self-attention in the future.

## 7. Conclusions

In this paper, we have presented a novel end-to-end and pixel-to-pixel segmentation network for retinal vessel segmentation. The proposed network is mainly implemented based on HRNet-shaped architecture with the addition of a strip attention module, which can maintain high-resolution representations through the whole process to obtain local spatial details and promote the fusion of features at different levels to obtain global semantic information. Extensive experiments on the DRIVE and STARE datasets demonstrate that the proposed method achieves promising segmentation performance over existing mainstream methods.

## Figures and Tables

**Table 1:** Description of datasets.

Dataset	Quantity	Train-Test Split	Resolution	Format	FoV Mask
DRIVE	40	20–20	565×584	.tiff	√
STARE	20	leave-one-out	700×605	.ppm	×

**Table 2:** Performance of models tested on DRIVE.

Method	ACC (%)	SE (%)	SP (%)	F1 (%)	AUC (%)
U-Net	95.31	75.37	98.20	81.42	97.55
Baseline	95.75	82.16	98.16	81.36	98.21
Proposed	96.16	82.68	98.47	84.27	98.64

**Table 3:** Performance of models tested on STARE.

Method	ACC (%)	SE (%)	SP (%)	F1 (%)	AUC (%)
U-Net	96.28	73.27	98.67	80.89	97.64
Baseline	96.64	84.62	98.48	84.23	98.42
Proposed	97.08	89.36	98.47	87.85	99.21

**Table 4:** Comparisons of the cross-training evaluation.

Dataset	Method	ACC (%)	SE (%)	SP (%)	AUC (%)
DRIVE (trained on STARE)	Jin et al. [17]	94.81	65.05	99.14	97.18
	Li et al. [20]	95.93	72.89	99.02	97.15
	Fathi et al. [36]	95.81	77.68	97.59	95.16
	Zhao et al. [37]	95.70	78.20	97.90	88.60
	Proposed	95.68	81.47	98.06	96.17
STARE (trained on DRIVE)	Jin et al. [17]	94.74	70.00	97.59	95.71
	Li et al. [20]	95.88	70.89	97.95	96.85
	Fathi et al. [36]	95.91	80.61	97.17	96.80
	Zhao et al. [37]	95.60	78.90	97.80	88.50
	Proposed	96.23	84.52	98.17	97.92

**Table 5:** Performance comparison with existing methods on DRIVE.

Method	Year	ACC (%)	SE (%)	SP (%)	F1 (%)	AUC (%)
GDF-Net [20]	2023	96.22	82.91	98.52	83.02	98.59
CSGNet [21]	2022	95.76	79.43	98.14	83.10	98.23
MPS-Net [22]	2021	95.63	83.61	97.40	82.87	98.05
Li et al. [38]	2021	95.68	79.21	98.10	-	98.06
HHNet [39]	2021	95.75	79.93	98.06	83.06	98.22
CSU-Net [40]	2021	95.65	80.70	97.82	82.51	98.01
MFI-Net [41]	2022	95.81	81.70	97.90	83.15	98.36
DCU-Net [42]	2022	95.68	81.15	97.80	82.72	98.10
MAGF-Net [43]	2023	95.78	82.62	97.83	83.07	98.19
Proposed	2023	96.16	82.68	98.47	84.27	98.64

**Table 6:** Performance comparison with existing methods on STARE.

Method	Year	ACC (%)	SE (%)	SP (%)	F1 (%)	AUC (%)
GDF-Net [20]	2023	96.53	76.16	99.57	80.22	98.89
CSGNet [21]	2022	97.10	83.57	98.62	85.42	99.10
MPS-Net [22]	2021	96.89	85.66	98.19	84.91	98.73
Li et al. [38]	2021	96.78	83.52	98.23		98.75
CSU-Net [40]	2021	97.02	84.32	98.45	85.16	98.25
MFI-Net [41]	2022	96.87	82.20	98.54	83.96	98.97
MAGF-Net [43]	2023	96.49	80.93	98.44	83.64	98.98
WA-Net [44]	2022	96.65	77.67	98.77	81.76	98.65
Bridge-Net [45]	2022	96.68	80.02	98.64	82.89	99.01
Proposed	2023	97.08	89.36	98.47	87.85	99.21

## Data Availability

The DRIVE dataset used in the experiments can be publicly obtained from the Grand Challenge website at https://drive.grand-challenge.org/DRIVE/ (accessed on 10 September 2023). The STARE dataset used in the experiments can be publicly obtained from the following website: https://cecas.clemson.edu/~ahoover/stare/ (accessed on 10 September 2023).
